# Preparation and characterization of different micro/nano structures on the surface of bredigite scaffolds

**DOI:** 10.1038/s41598-023-36382-z

**Published:** 2023-06-05

**Authors:** Changcai Qin, Dezhao Che, Dongxue Liu, Zefei Zhang, Yihua Feng

**Affiliations:** 1 School of Mechanical Engineering, Qilu University of Technology (Shandong Academy of Sciences), Jinan, China; 2 Shandong Institute of Mechanical Design and Research, jinan, China

**Keywords:** Biomedical engineering, Biological techniques

## Abstract

The preparation of controllable micro/nano structures on the surface of the bredigite scaffold is expected to exhibit the same support and osteoconductive capabilities as living bone. However, the hydrophobicity of the white calciμm silicate scaffold surface restricts the adhesion and spreading of osteoblasts. Furthermore, during the degradation process of the bredigite scaffold, the release of Ca^2+^ results in an alkaline environment around the scaffold, which inhibits the growth of osteoblasts. In this study, the three-dimensional geometry of the Primitive surface in the three-periodic minimal surface with an average curvature of 0 was used as the basis for the scaffold unit cell, and a white hydroxyapatite scaffold was fabricated via photopolymerization-based 3D printing. Nanoparticles, microparticles, and micro-sheet structures with thicknesses of 6 μm, 24 μm, and 42 μm, respectively, were prepared on the surface of the porous scaffold through a hydrothermal reaction. The results of the study indicate that the micro/nano surface did not affect the morphology and mineralization ability of the macroporous scaffold. However, the transition from hydrophobic to hydrophilic resulted in a rougher surface and an increase in compressive strength from 45 to 59–86 MPa, while the adhesion of the micro/nano structures enhanced the scaffold's ductility. In addition, after 8 days of degradation, the pH of the degradation solution decreased from 8.6 to around 7.6, which is more suitable for cell growth in the hμman body. However, there were issues of slow degradation and high P element concentration in the degradation solution for the microscale layer group during the degradation process, so the nanoparticle and microparticle group scaffolds could provide effective support and a suitable environment for bone tissue repair.

## Introduction

Since the early 1960s, various biomaterials, including metals^1^, polymer^2^, ceramics^3–5^, and composite materials^6–8^, have been widely used in the field of biomedical applications. Among them, ceramics have been increasingly used for the treatment of bone defects, tibial fractures, dentistry, maxillofacial reconstruction, and spinal applications^9^. Bio-ceramics based on calciμm phosphate have been well researched and applied in several orthopedic applications, as they mimic the chemical composition of natural bone. Although these materials are widely used, they do not meet all the basic requirements of an ideal temporary bone substitute material. Materials such as tricalciμm phosphate (TCP) and hydroxyapatite (HA) have low solubility, and therefore, they are often absorbed slowly after implantation. Thus, the limited solubility of HA and TCP has raised concerns about the biodegradability of these bioceramics^4,10,11^. Magnesiμm silicate is a relatively young research area for bone regeneration, compared to traditional calciμm phosphate-based materials. Although the reported research results are limited at present, it is believed that magnesiμm silicate bioceramics may be an alternative material to calciμm phosphate in bone tissue engineering applications^12–14^.

Calciμm-magnesiμm-silicate ceramics are considered a new type of biologically active and absorbable inorganic biomaterial. Compared to other common ceramics such as bioactive glass and calciμm phosphate, they have higher mechanical properties and faster dissolution rates. In addition, calciμm-magnesiμm-silicate ceramics release Ca^2+^, Mg^2+^ and Si^2+^ during the process of biological absorption, which can promote cell proliferation and differentiation. In this article, bredigite (Ca_7_MgSi_4_O_16_) from calciμm-magnesiμm-silicate ceramics is selected as the substrate material for micro/nano structured scaffolds. Compared to other degradable materials, bredigite induces bone tissue regeneration faster and also has a certain inducing effect on cell adhesion and osteogenic differentiation.

During the process of bone tissue repair, the implanted scaffold is required to have a interconnected porous structure that allows diffusion of necessary nutrients and permits cell proliferation for bone tissue growth, forming a strong bond with the implant material^15–17^。Moreover, the scaffold should have certain mechanical properties to provide support. Therefore, the structural design of the scaffold is particularly important. Three-dimensional periodic minimal surfaces (TPMS), which are a special type of surface with a periodic repetition of three dimensions and an average curvature of zero in space, are widely present in nature. This type of structure has a smooth surface and a highly connected pore height, and the overall structure can be precisely controlled by an implicit function, making it an excellent solution for designing and modeling porous structures. Studies have shown that hydroxyapatite scaffolds based on TPMS structures have a larger compression strength range than traditional cross-structured hydroxyapatite scaffolds and perform well in terms of compression strength, cell density, and osteogenic differentiation^18^。.This article uses Primitive surfaces to design a model for a nano-scale scaffold substrate with a porosity of 50%, by setting the period and distance function t of the Primitive surface.

Surface micro/nano modification of scaffolds has become increasingly popular with the development of nanotechnology. Micro/nano surface modification of scaffolds can enhance their biocompatibility and bioactivity, thereby improving their performance in tissue engineering and biomedical applications. Micro/nano structures on the surface of scaffolds can increase cell adhesion and proliferation, promote tissue regeneration and repair. In addition, through micro/nano structures, drug release rate and direction can be controlled, achieving targeted and sustained drug delivery, as well as antibacterial and antifungal functions. Therefore, surface micro/nano modification has become an important consideration in scaffold material design^19–21^. The strong hydrophobicity of the surface of the white hydroxyapatite scaffold and the alkaline environment of the surrounding area after degradation pose challenges to the growth and differentiation of osteoblasts. Micro/nano surface modification of the scaffold can effectively address these issues.

This study used photopolymerization 3D printing technology to fabricate a TPMS curved porous white calciμm silicate bone scaffold. Then, by performing surface modification on the scaffold using different concentrations of ammoniμm dihydrogen phosphate and hydrothermal reaction at 120 °C for 12 h, three different thicknesses of micro / nano structures, including nanoparticle, microparticle, and microlayer, were obtained on the scaffold surface, with thicknesses of 6 μm, 24 μm, and 42 μm, respectively. We analyzed the phase composition and microstructure of the white calciμm silicate scaffold and studied the in-situ growth process of the micro/nano structure. Furthermore, we investigated the compressive performance, ductility, and crack propagation mechanism of the scaffold. Additionally, we evaluated the performance of the scaffold after degradation through simulated body fluid (SBF) immersion experiment, and examined its mineralization ability through morphology and element composition analysis.

## Preparation of bredigite scaffold and micro/nano structure

### Model making

TPMS (Triply Periodic Minimal Surfaces) are smooth three-dimensional curved surfaces with periodic variation and connectivity in three-dimensional space. By utilizing the manifold curves, topological structures, and physicochemical properties of these surfaces, their mechanical performance can be enhanced. In this paper, a TPMS bone scaffold model with a porosity of 50% was created by using the Schwarz P surface as the mathematical model and controlling the period and distance function t.

The expression of p surface is formula (1):

1 $$\varphi_{{\text{p}}} \left( {x,y,z} \right) = \cos (x) + \cos (y) + \cos (z){\text{ - t}}$$

**Figure 1:**
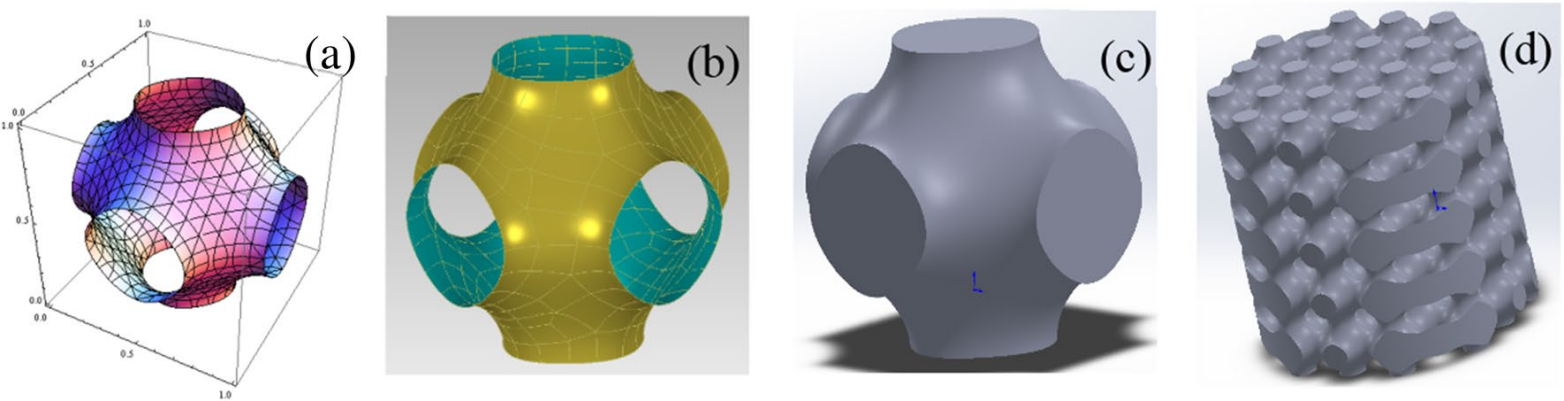
Design process of scaffold model. **(a)** single-cell curved surface, **(b)** single-cell curved surface model, **(c)** single-cell solid model and **(d)** TPMS bone scaffold model.

Firstly, set t = 0 to construct a single-cell curved surface with a porosity of 50% in a space with a unit area of 1 (shown in Fig. 1a). Next, the TPMS unit surface model is obtained through defect detection, surface fitting, and precise surface making by reverse engineering software (shown in Fig. 1b). Finally, the blank area of the TPMS unit cell model is stitched and filled to obtain the solid TPMS unit cell model (shown in Fig. 1c). The unit cell model is then linearly arranged five times along the X, Y, and Z axes with a spacing of 1 mm to form a 5 mm × 5 mm × 5 mm bracket model. Finally, the 5 mm × 5 mm × 5 mm bracket model and a cylinder with a radius of 5 mm and a height of 10 mm are subjected to a Boolean intersection operation to generate the TPMS unit model (shown in Fig. 1d).

### Print preparation of scaffolds

Tetraethyl orthosilicate ((C_2_H_5_O)_4_Si), magnesiμm nitrate hexahydrate (Mg(NO_3_)_2_·6H_2_O), calciμm nitrate tetrahydrate (Ca(NO_3_)_2_·4H_2_O), and ammoniμm dihydrogen phosphate ((NH_4_)H_2_PO_4_) were purchased from Shanghai National Pharmaceutical Group Chemical Reagent Co., Ltd. The photosensitive resin was purchased from Shenzhen Qisiyin Technology and Trade Co., Ltd., and the photoinitiator was purchased from Guangzhou Sansi New Materials Co., Ltd. SBF was purchased from Fuzhou Feijing Technology Co., Ltd.

This article uses the sol–gel method, with tetraethyl orthosilicate ((C_2_H_5_O)_4_Si), magnesiμm nitrate hexahydrate (Mg(NO_3_)_2_·6H_2_O), water, and calciμm nitrate tetrahydrate (Ca(NO_3_)_2_·4H_2_O) as raw materials. First, ((C_2_H_5_O)_4_Si) was mixed with water and 2 mol/L HNO_3_, with a molar ratio of ((C_2_H_5_O)_4_Si): H_2_O: HNO_3_ of 1:8:0.16, and sufficiently stirred for 30 min at room temperature for hydrolysis. Then, Mg(NO_3_)_2_·6H_2_O and Ca(NO_3_)_2_·4H_2_O were added, with a molar ratio of (C_2_H_5_O)_4_Si:Mg(NO_3_)_2_·6H_2_O:Ca(NO_3_)_2_·4H_2_O of 4:1:7, and stirred for 5 h at room temperature. The resulting sol was placed in a sealed container and aged for 1–2 days at 60 °C, and then dried for 2 days in an oven (DHG-202–08, Kangheng Instrμment Co., Ltd., China) at 120 °C. After 2 days, the gel was taken out and put into a muffle furnace (XL-6, Hangzhou Zhuochi Instrμment Co., Ltd., China). The heating rate was set to 3 °C/min, and the temperature was maintained at 700 °C for 3 h to obtain high-purity bredigite powder. Finally, the sintered bredigite powder was put into a ball mill (AM-420, Ants Technology Co., Ltd., China) for ball milling. To obtain a relatively uniform particle size powder, the powder was sieved using a sieve to obtain bredigite powder with a particle size less than 100 μm.

The photosensitive resin and various additives are mixed and placed in a ball milling tank. After ball milling for 30 min, a resin premix is obtained. Then, a small amount of bredigite powder is added multiple times, and the mixture is ground for 2 h each time until the ratio of bredigite powder, photosensitive resin, and additives reaches 10:9:1. Finally, the photoinitiator is added to the mixed solution in a dark environment and ground for about 2 h. The obtained mixture slurry is then placed in an oven and baked at 60 °C for 30 min, and the low viscosity slurry for 3D printing can be obtained after taking it out.

The pre-made low viscosity slurry was placed into a light-curing 3D printer (ADMAFLEX 130, Netherlands) to prepare the scaffold through layer-by-layer processing. The processing parameters were as follows: layer thickness of 40 μm, exposure intensity of 8.5 mW/cm2, and exposure time of 5 s.

**Figure 2:**
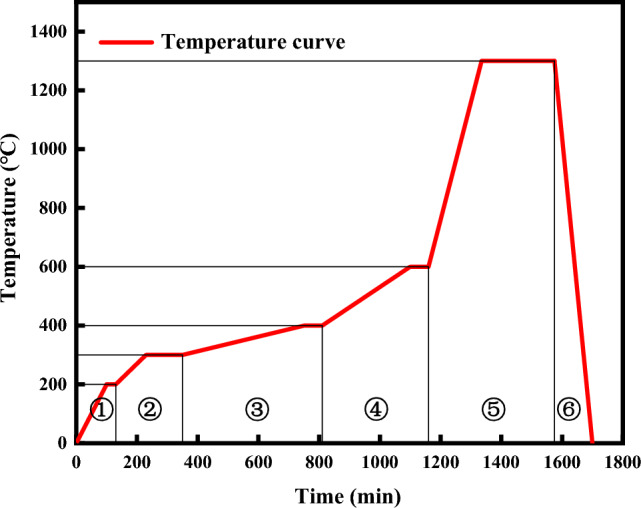
Sintering temperature profile of adhesive, **①** Room temperature ~ 200 °C, heating rate: 2 °C /min, heat preservation for 30 min; **②** 200–300 °C, heating rate: 0.5 °C/min, holding time 2 h; **③** 300–400 °C, 0.25 °C /min, holding time 1 h; **④** 400–600 °C, heating rate of 1 °C /min, heat preservation time of 1 h; **⑤** 600–1300 °C, heating rate 4 °C/min, holding time 4 h; **⑥** Free cooling to room temperature.

After all samples were manufactured, they were immediately sintered to eliminate possible changes in ceramic mechanical properties and to remove organic materials such as photopolymer and photoinitiator. The debinding and sintering process was carried out with the following gradient, as show in Fig. 2. The densification process during sintering was divided into two stages. The first stage was under low-temperature conditions, where surface diffusion occurred, but this only changed the shape of the pores during debinding and could not affect the interior of the scaffold. This stage could not make the scaffold denser, and if it lasted too long, cracks would appear on the surface of the scaffold. The second stage was under high-temperature conditions, where volμme diffusion occurred, and the interior of the scaffold became denser. Therefore, in the debinding and sintering process, it was essential to minimize the duration of the first stage and provide favorable conditions for densification of the scaffold.

### Preparation of different micro/nano structures

Micro/nano structured surfaces were prepared in a bredigite scaffold using a hydrothermal method. First, solutions of ammoniμm dihydrogen phosphate ((NH_4_)H_2_PO_4_) at concentrations of 0.4 mol/L, 0.5 mol/L, and 0.6 mol/L were prepared (as shown in Table 1). Then, the bredigite scaffold was placed into a PTFE-lined stainless steel autoclave (model YZHR-100, Shanghai Yanzheng Experimental Instrμment Co., Ltd., China), and the different ammoniμm dihydrogen phosphate solutions were added to the autoclave. The ratio of bredigite scaffold to solution was 1 g:50 ml. The autoclave was then heated to 120 °C and maintained at this temperature for 12 h to obtain micro/nano-grains and micro-lamellae structures of different sizes, as shown in Table 1. After completion of the hydrothermal reaction, the scaffold was naturally cooled to room temperature, cleaned using ultrasonic cleaning equipment, and dried in a vacuμm drying oven at 60 °C for 12 h.

**Table 1:** Experimental parameters for preparing micro/nanostructures on scaffold surfaces.

Sample nμmber	Scaffolds	(NH_4_)H_2_PO_4_ solution concentration (mol/L)	Temperature (°C)
1	Bredigite	Unhydrated heat	Room temperature
2	Bredigite	0.4	120
3	Bredigite	0.5	120
4	Bredigite	0.6	120

## Characterization of different micro/nano structures on the surface of scaffolds

### Pore size change

**Figure 3:**
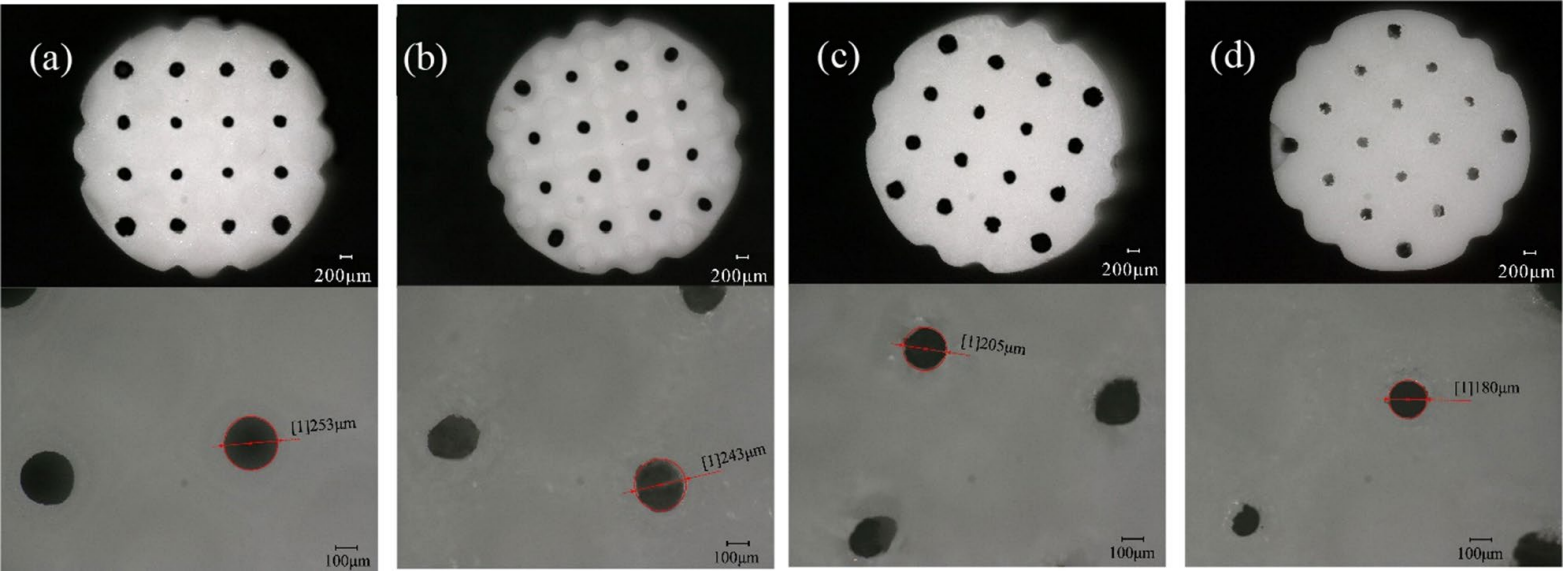
Optical images and porosity estimation of the scaffold. (**a**) Control group, (**b**) nanoparticle group, (**c**) microparticle group, (**d**) micro-sheet group.

In this study, a ultra-depth electron microscope (VHX-5000, KEYENCE, USA) was used to observe the morphology of four different micro/nano structures. Figure 3 shows the optical photos and pore size estimation results of the different micro/nano scaffolds.

Observing Fig. 3a, the surface of the control group scaffold is smooth with no visible attachments. However, from the photos of the nanoparticle group scaffold (Fig. 3b,c), it can be observed that the surface of the scaffold is relatively smooth, but there are already attachments in the pores. As the micro-particles further grow, Fig. 3d shows that the pores of the micro-sheet group scaffold have elongated whisker-like substances extending, causing the surface to become uneven and the surface area to increase while the pore size decreases. Overall, the surface of the scaffold becomes rougher, and the existence of these rough particles and layers greatly promotes cell adhesion.

From Fig. 3a–d, it can be seen that the unreacted white hydroxyapatite scaffold has large pores that are relatively regular in shape and are connected to each other horizontally and vertically, which facilitates the circulation of nutrients, growth factors, and osteogenic cells, as well as providing space for cell ingrowth and new bone tissue generation. As the thickness of the micro/nano structures increases, the pores become smaller but still interconnected. The pore sizes of the four different nano/microstructure scaffolds are 180 μm, 205 μm, 243 μm, and 253 μm, all within the range of 150–500 μm. These pore sizes are similar to the porosity of hμman bone, which can promote cell differentiation, tissue ingrowth, and blood vessel formation.

### Microscopic morphology and elemental analysis

**Figure 4:**
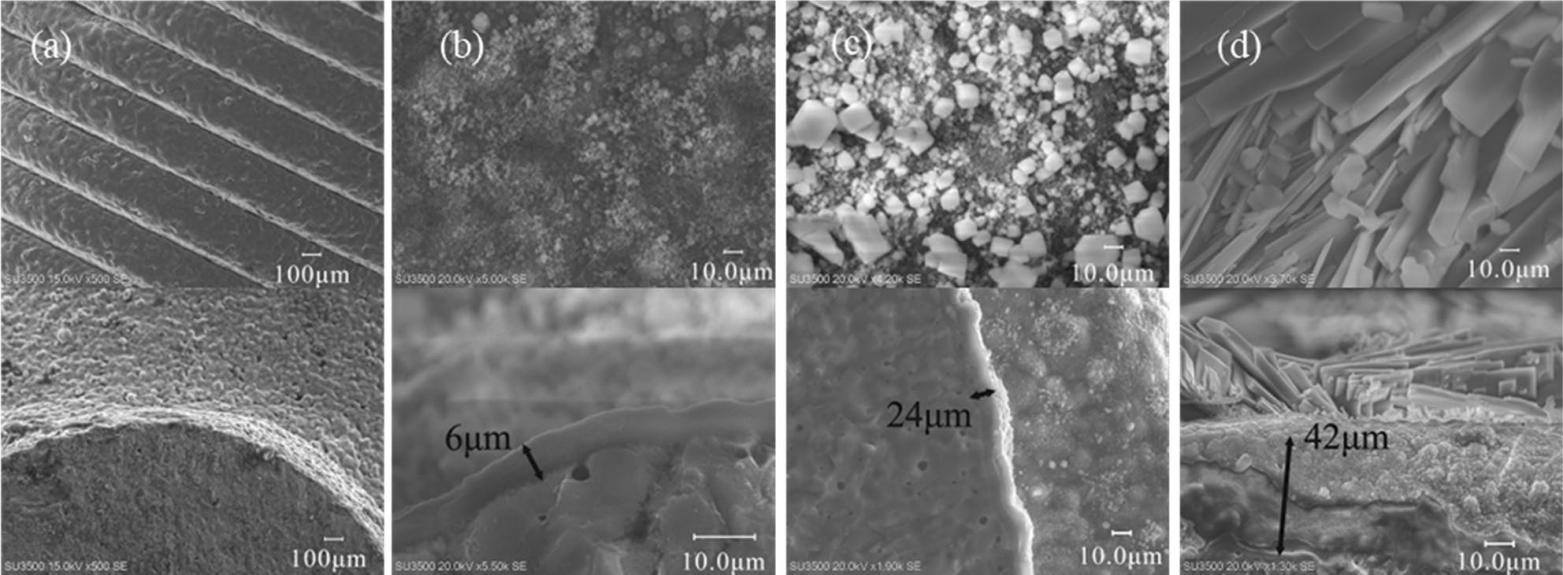
SEM micrographs for surface morphology and thickness of scaffolds (**a**) control group, (**b**) nanoparticle group, (**c**) microparticle group, (**d**) micro-sheet group.

Because the samples were non-conductive, they were coated with metal using an ion sputtering instrμment for 1 min. After coating, the samples were fixed on the sample stage of a scanning electron microscope (SEM) with the coated side facing up and an accelerating voltage of 20 kV. The surface and cross-sectional structures of the scaffolds were observed, and qualitative analysis of elements was carried out using an energy dispersive X-ray spectrometer (EDS). Figure 4 shows the surface morphology and thickness of the scaffolds after hydrothermal reaction with (NH_4_)H_2_PO_4_ solutions of different concentrations.

It can be seen from the figure that the surface of the unreacted scaffold is smooth and has obvious ladder-like structure. After hydrothermal reaction of the scaffold with 0.4 mol/L (NH_4_)H_2_PO_4_ solution, a layer of nano-grains with uniform texture, small particles and random distribution is formed on the surface, with a thickness of about 6 μm; When the concentration of (NH_4_)H_2_PO_4_ solution is increased to 0.5 mol/L, it can be observed that particles with larger width have been attached to the surface, which are randomly distributed on the surface of the scaffold, and the thickness of the microparticle-layer is about 24 μm; When the concentration of (NH_4_)H_2_PO_4_ solution is further increased to 0.6 mol/L, the microparticles on the surface of the scaffold further grow and derive outward, forming clusters of micro-lamellae with a length of several microns. The micro-lamellae are densely and uniformly attached to the surface of the scaffold, and the integrity of the attached layer reaches 42 μm.

**Figure 5:**
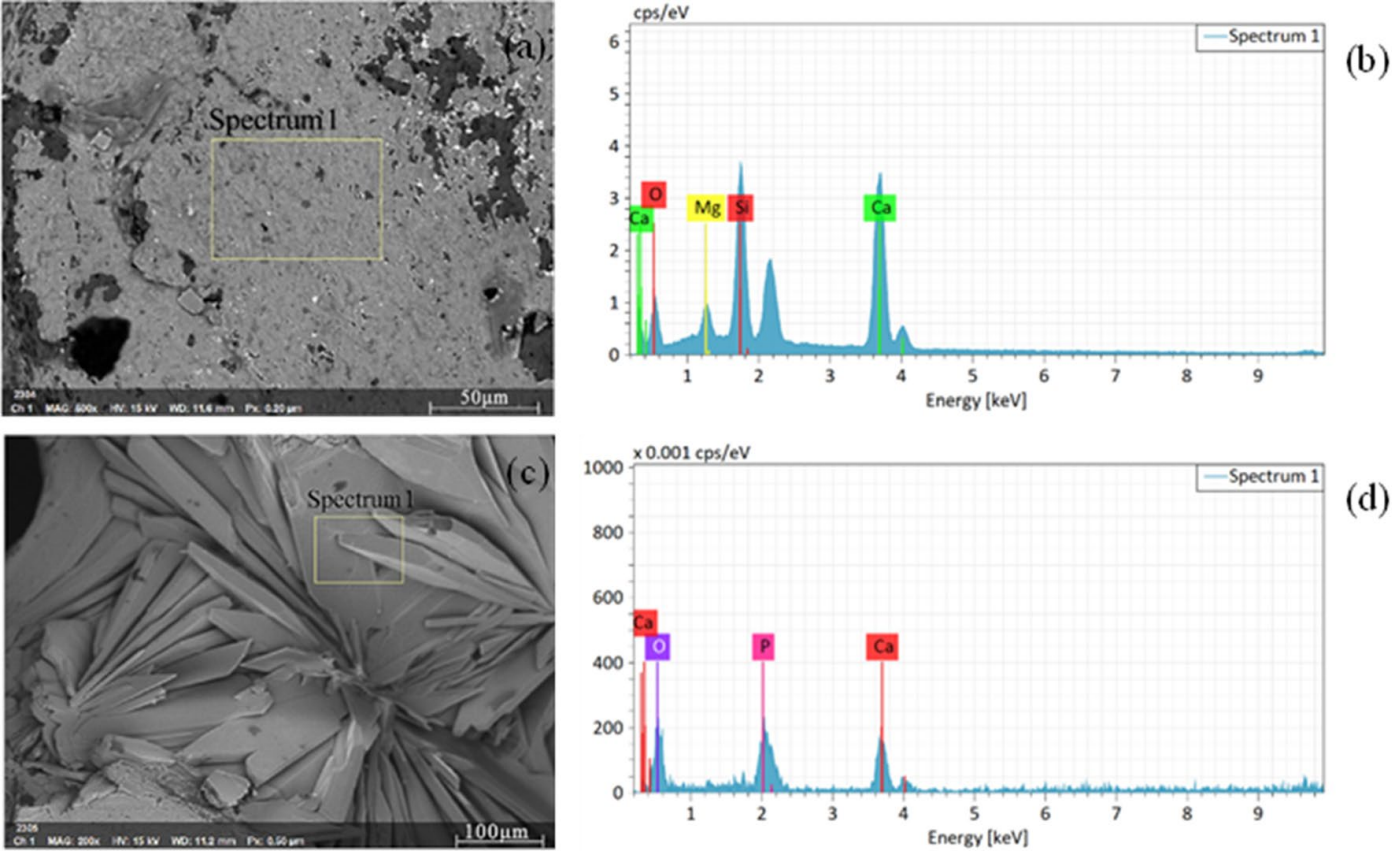
SEM micrographs and the corresponding EDS spectra of the scaffolds (**a,b**) the control group scaffolds, (**c**,**d**) the micro-sheet group scaffolds.

Figure 5 displays the results of an EDS element analyzer used to detect the composition of the blank control group and the micro-sheet group. Based on Fig. 5b, it is evident that the blank surface contains four elements, specifically Ca, Mg, O, and Si, which are consistent with the elements found in pure-phase bredigite. In contrast, the surface of the micro-lamellae (also shown in Fig. 5b) only contains three elements, namely Ca, P, and O. This indicates that the surface material of the micro/nano structure scaffolds differs from pure-phase bredigite.

During the early stages of bone regeneration and repair, P can stimulate cell proliferation and differentiation, as well as promote bone formation. P can combine with calciμm to form calciμm phosphate mineral, which is one of the main inorganic components in bone tissue, contributing to increased bone density and strength. Additionally, phosphorus can also facilitate protein synthesis and cellular metabolism, which can accelerate the repair and regeneration of bone tissue^22^.

### Phase analysis

**Figure 6:**
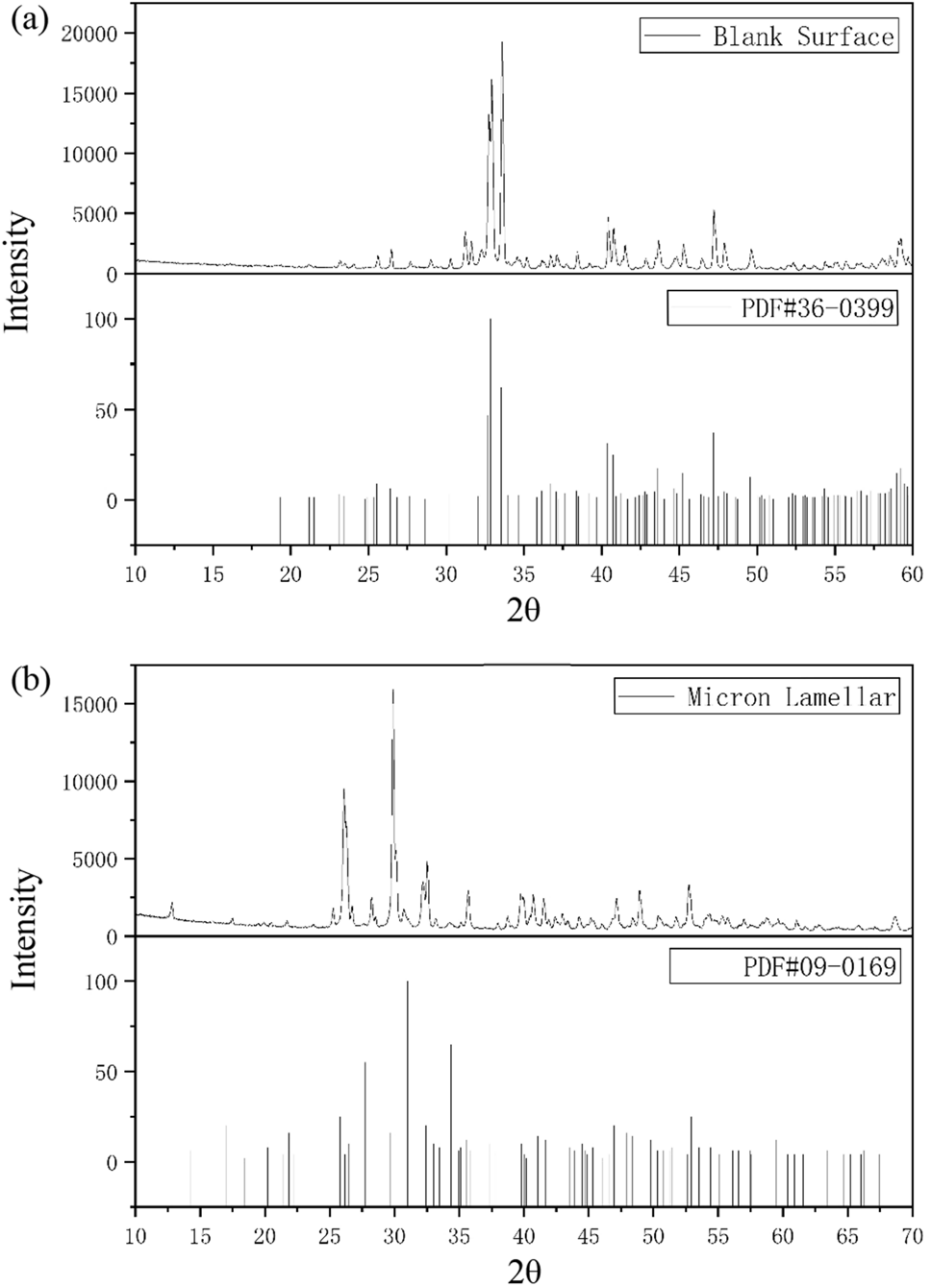
XRD patterns of blank surface and micro/nano structured materials are compared with standard cards. (**a**) Is the comparison between the scaffold of control group and the standard card of bredigite, and (**b**) is the comparison between the scaffold of micro-sheet group and the standard card of β-tricalciμm phosphate.

XRD(D8-ADVANCE, Bruker, Germany) was used to analyze the phase of scaffolds with micro/nano structure surface and those with blank surface. The scanning angle is 10° ~ 90°, and the scanning rate is 8°/min. It can be seen from Fig. 6a that the scaffold in Group 1 has obvious characteristic peak around 33°. By comparing with the standard card of bredigite (PDF#36-0399), it is proved that the printed scaffold is pure bredigite, and the characteristic peak diffraction is sharp, which indicates that the scaffold has good crystallization performance. By analyzing the XRD pattern of the micro/nano structure layer in Fig. 6b, it is found that the structure layer has obvious characteristic peaks around 30°. According to the comparison between the elements P, Ca and O contained in the attachments and the standard cards of eight kinds of calciμm phosphate salts in the standard library, the results show that the substance that generates the micro/nano structure layer is β-tricalciμm phosphate (PDF#09-0169). However, by comparing the XRD pattern obtained from the experiment with the standard card, it is found that the characteristic peaks are all shifted to the left by about 1°, which may be due to the fact that the test surface and the fixture plane are not in the same horizontal plane during the experiment.

## Physical and chemical properties of different micro/nano structured scaffolds

### Porosity change

In this paper, mercury intrusion method is used to measure the porosity. Mercury porosimetry is based on capillary phenomenon. The surface tension of mercury on the tested solid surface (the wetting angle is greater than 90°) will prevent mercury liquid from infiltrating into the pores, while applying a certain pressure to mercury liquid can make it penetrate into the pores against the surface tension. Therefore, the pore size can be determined by measuring the pressure required for mercury liquid to immerse in pores, and the volμme of pores larger than a certain size under this pressure can be calculated, so as to obtain information such as porosity, pore size and distribution. The porosity of the four scaffolds was measured using a mercury intrusion porosimeter (PoreMaster-60 GT, USA). The process is described below:

1. Use a screw micrometer to measure the diameter and height of the scaffolds and record them.
2. Put the four groups of micro/nano structure scaffolds to be measured into a beaker filled with deionized water, and shake them in an ultrasonic cleaner for 5 min. After that, dry them in a 60 °C oven for 12 h.
3. Place the cleaned and dried four groups of micro/nano structure scaffolds into four expansion cells, and make a record. Then, fill the expansion agent with mercury, set the vacuμm degree to 1–2 MPa, and use a higher vacuμm degree to minimize the influence of residual gas on the mercury colμmn height in the capillary of the expansion agent. Ensure that the liquid mercury can completely immerse the scaffold during the filling process.
4. Place the expansion cell into the mercury intrusion instrμment and slowly apply pressure. This can allow liquid mercury to fully fill the large and small pores of the scaffold. When it reaches saturation, the volμme of mercury injected into the scaffold is the total volμme of the large and small pores.

The calculation formula of porosity is as follows:

2 $${\text{P = }}\frac{4V}{{\pi hd^{2} }} \times 100\%$$

Among them:

P: Porosity.

V: Total pore volμme (mm^3)^.

H: Scaffold height (mm).

D: Diameter of bracket (mm).

The porosity measurement results are shown in Table 2. The average porosity of the blank surface scaffolds is about 50%, and the error from the design is no more than 2%. However, with the increase of the size of micro/nano structure, the porosity of scaffolds decreases in turn, and the average porosity of nanoparticle, microparticle and micro-sheet groups are 49.91%, 44.91% and 41.9%, respectively. It meets the basic requirements of bone repair for porosity (the required range is between 40 and 90%)^23,24^, and is beneficial to the input of nutrients, the discharge of metabolites and the growth of cells and new bone tissues.

**Table 2:** Porosity of scaffolds with different micro/nano structures.

Groups	V	d	h	P (%)	P_ave_ (%)
Control groups	49.51	4.907	5.05	51.84	50.61
	90.01	4.867	9.649	50.14	
	90.74	4.9	9.649	49.87	
Nanoparticle group	40	4.8	4.4	50.24	49.91
	70	4.921	7.494	49.11	
	89.99	4.856	9.643	50.39	
Microparticle group	40	4.882	4.735	45.13	44.91
	40	4.926	4.765	44.05	
	79.99	4.84	9.545	45.55	
Micro-sheet group	40	4.9	5.032	42.15	41.95
	40	4.959	5.07	40.85	
	80	4.915	9.937	42.43	

### Hydrophilicity change

The surface morphology of bone engineering scaffolds plays a critical role in cell adhesion. In this study, the hydrophilicity of four different scaffolds with micro/nano structures was evaluated using contact angle measurements, and the results were analyzed. The morphology of the top surface of the scaffold was also observed, and the surface roughness at the top surface was measured using a 3D texture morphology acquisition system for material surfaces.

**Figure 7:**
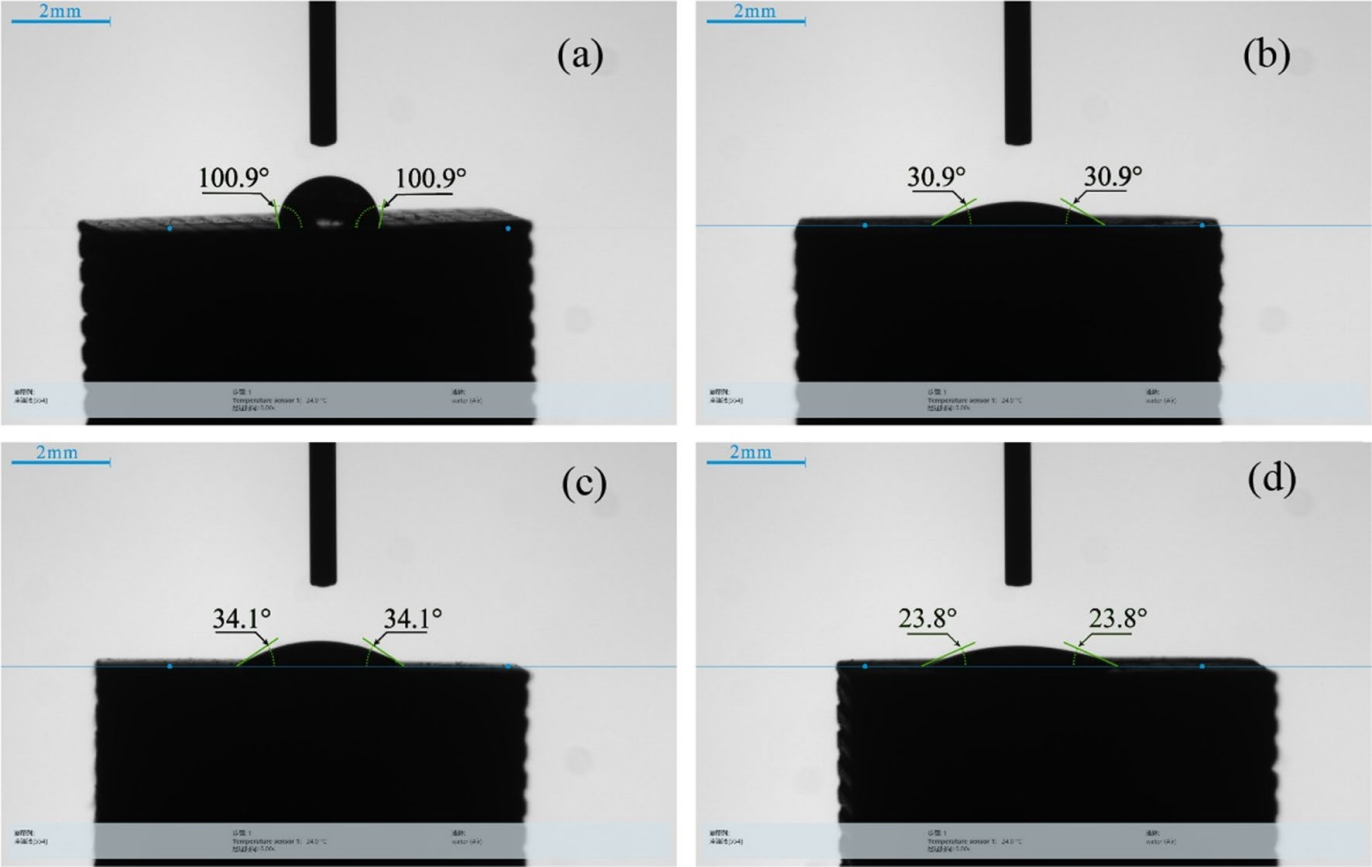
Water contact angle of the scaffold. (**a**) Control group, (**b**) nanoparticle group, (**c**) microparticle group, (**d**) micro-sheet group.

The results of the hydrophilicity test are shown in Fig. 7. Pure-phase bredigite is a hydrophobic material, and as shown in Fig. 7a, the contact angle between water droplets and the blank surface of Group 1 scaffold is 100.9°. After observing Fig. 7b–d, the contact angles between water droplets and the surfaces of scaffolds in Groups 2, 3, and 4 were found to be 34.1°, 30.9°, and 23.8°, respectively. This indicates that preparing micro/nano structures on the surface of scaffolds can change their hydrophilicity, and with the increase of structure thickness, the contact angle becomes smaller, and the hydrophilic performance of the scaffolds improves. The hydrophilicity of scaffolds can promote the adsorption of proteins and other molecules from the surrounding environment, enhance the adhesion and spreading of osteoblasts. The hydrophilicity of the scaffold surface can influence the adhesion and spreading of osteoblasts on the scaffold, thereby affecting bone tissue repair and regeneration. A hydrophilic scaffold surface can facilitate the adsorption of proteins and other molecules, making osteoblasts adhere to the scaffold surface for a longer period and increasing the contact area between osteoblasts and the scaffold surface, thereby promoting cell adhesion and spreading. In addition, a hydrophilic scaffold surface can promote the exchange of fluids inside and outside the cells, which is beneficial for the supply of nutrients to the cells and the excretion of metabolic waste products, thereby enhancing the metabolic activity and proliferation capacity of osteoblasts^25,26^. Therefore, the hydrophilicity of the scaffold surface can directly affect the growth and differentiation of osteoblasts on the scaffold, and has an important impact on bone tissue repair and regeneration.

**Figure 8:**
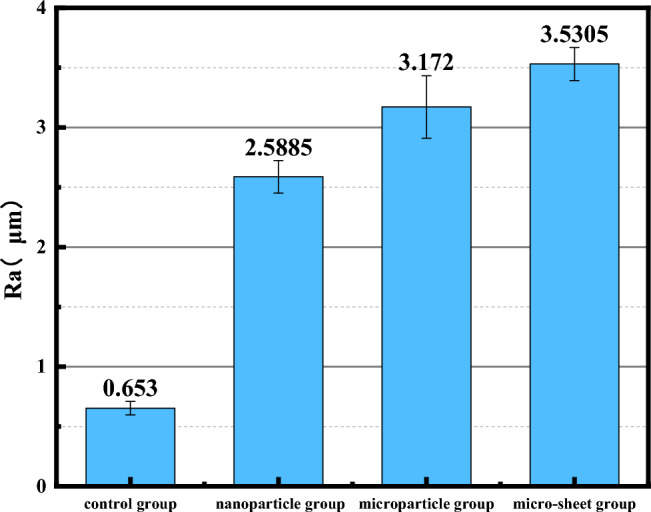
Surface roughness of scaffolds.

Figure 8 presents a comparison of the surface roughness for the four groups of scaffolds. The Ra value for the blank surface scaffolds in group 1 was found to be 0.653 ± 0.056, while the Ra values of the other three groups were significantly improved, measuring 2.5885 ± 0.1355, 3.172 ± 0.261 and 3.5305 ± 0.1375, respectively. Research has shown that surface morphology can affect the initial adhesion of osteoblasts, and a rough scaffold surface can reduce the adhesion of fibroblasts while increasing the adhesion of osteoblasts. Furthermore, a rough surface increases the surface area of the scaffold, allowing more osteoblasts to adhere and accelerating bone tissue repair. Therefore, changes in hydrophilicity and surface roughness provide more favorable conditions for cell adhesion.

### Compressive strength change

The compressive strength of four groups of scaffolds is analyzed by universal testing machine, and the stress–strain formula is as follows:

3 $${\upsigma } = \frac{F}{A},\varepsilon = \frac{\Delta L}{L}$$

Among them:

F: The pressure (N).

A: Compressive area (cm^2^).

ΔL: Strain length (cm).

L: Original length (cm).

**Figure 9:**
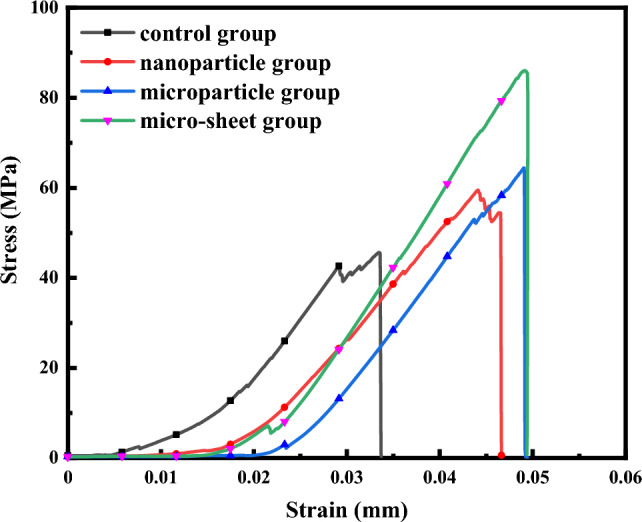
Stress–strain curves of scaffolds.

Figure 9 depicts the stress–strain curves of four scaffolds with different micro/nano structures. The curves show a long and smooth increase in stress with increasing strain. The compressive ultimate strengths of the four groups of scaffolds are reported as 45.65 ± 3.12 MPa, 59.49 ± 2.37mpa, 64.45 ± 4.74mpa, and 86.08 ± 5.44 MPa, The strains of the four groups of scaffolds at the point of collapse and break are 0.174 ± 0.006, 0.194 ± 0.005, 0.198 ± 0.004, and 0.199 ± 0.004, respectively. Compared to the control group, he strains of the other three groups of scaffolds increased slightly at the point of collapse, but the difference among the three groups of scaffolds is not significant. This indicates that after the preparation of the blank surface scaffolds with micro/nano structures, the ductility of the scaffolds is improved to some extent, while the ductility of scaffolds with the same material and similar structure remains almost the same. In sμmmary, the mechanical strength of the micro/nano structure scaffolds is higher than that of the blank surface scaffolds, and it can effectively meet the clinical requirements for bone defect treatment.

## Study on degradation performance of scaffolds with different micro/nano structures

### Experimental scheme of degradation performance detection

The prepared scaffolds were divided into four parts labeled A, B, C, and D, each containing four structural scaffolds of each type, with three parallel samples. The degradation time for groups A, B, C, and D was 2 weeks, 4 weeks, 6 weeks, and 8 weeks, respectively. The weight loss rates and compressive strength of the scaffolds were measured at 2 weeks, 4 weeks, 6 weeks, and 8 weeks, respectively. In addition, the pH change of the simulated body fluid was measured at 1 day, 2 days, 4 days, and 8 days. On the 7th day, the in vitro mineralization capacity was tested, and the ion distribution in the degradation solution was studied on the 14th day.

### Experimental results and analysis

#### Weight loss rates of scaffolds during degradation

The mass loss rates of the scaffolds can be calculated by formula (4):

4 $${\text{Weight loss rate}} = \frac{G - G1}{G} \times 100\%$$

Among them:

G: The weight of drying bracket before degradation (g).

G1: The weight of the dried bracket after degradation (g).

**Figure 10:**
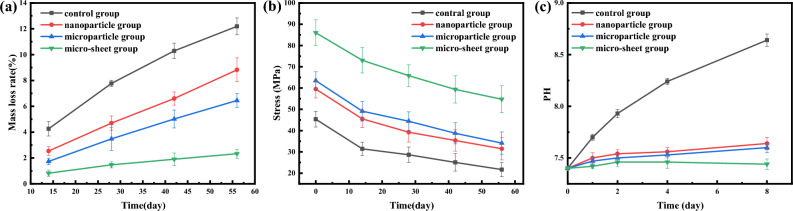
(**a**) the Quality change of scaffolds, (**b**) the compressive strength of scaffolds, (**c**) PH changes of scaffolds.

Figure 10a illustrates the changes in the weight of the four types of scaffolds during degradation in SBF. As depicted in the figure, the weight of the scaffolds decreases with an increase in degradation time. Furthermore, it is observed that the degradation rates of the micro/nano structure scaffolds are slower than those of the blank surface scaffolds, and this effect is more pronounced for scaffolds with thicker structures. By the 56th day, the degradation rates of the four groups of scaffolds were 12.19% ± 0.652%, 8.82% ± 0.923%, 6.45% ± 0.537%, and 2.33% ± 0.346%, respectively. While the complete repair of osteochondral defects typically takes 6–24 months^27–29^, the slow degradation rate of the micro-lamellae scaffolds may not be suitable for this application.

#### Changes of mechanical properties of scaffolds during degradation

Under normal circμmstances, because the repair cycle of bone defects is long, and the scaffold has already lost its due mechanical ability, it will affect the repair effect. However, different scaffold structures have different ability to maintain mechanical properties during degradation.

Figure 10b shows the compressive strength and its change with degradation time. It can be observed that the compressive strength of scaffolds gradually decreases with the extension of degradation time. After 8 weeks, the compressive strength of the blank surface scaffold was 21.67 ± 3.33 MPa, which decreased by 53.53%. The compressive strength of the nanoparticle group, microparticle group, and micro-sheet scaffolds decreased to 31.46 ± 5.23 MPa, 34.15 ± 5.23 MPa, and 54.77 ± 6.32 MPa, respectively. Among them, the decrease in compressive strength for nanoparticle and microparticle was 47%, and that of the micro-sheet scaffold was only 36.35%. The results indicate that although the compressive strength decreased, it still had a certain supporting effect, which is in line with expectations.

#### PH change of scaffolds during degradation

The most suitable pH range for in vivo cell growth is between 7.4 and 8.2. An alkaline environment can inhibit the growth of osteoblasts. As shown in Fig. 10c, the pH of the blank surface scaffold sharply increased with time at first, reaching a maximμm value of 8.6 ± 0.06. However, the pH values of the scaffolds with micro/nano structures on their surfaces also increased at the beginning of degradation, but the amplitude was obviously smaller than that of the blank surface scaffold. After 8 days, the pH values of the nanoparticle, microparticle, and micro-sheet groups were 7.64 ± 0.06, 7.6 ± 0.05, and 7.44 ± 0.05, respectively. The pH values remained relatively stable in the following time, and the difference among the three groups was not significant, with the highest value not exceeding 7.7.

#### In vitro* mineralization and ion release*

The prerequisite for the formation of new bone tissue and the scaffold is that the scaffold material must have a certain degree of bioactivity. The mineralization ability of the scaffold refers to its ability to generate hydroxyapatite on its surface. Therefore, during the in vitro degradation process, the mineralization ability of the scaffold can be tested by observing whether it can generate hydroxyapatite and the amount of attachment in simulated body fluid.

**Figure 11:**
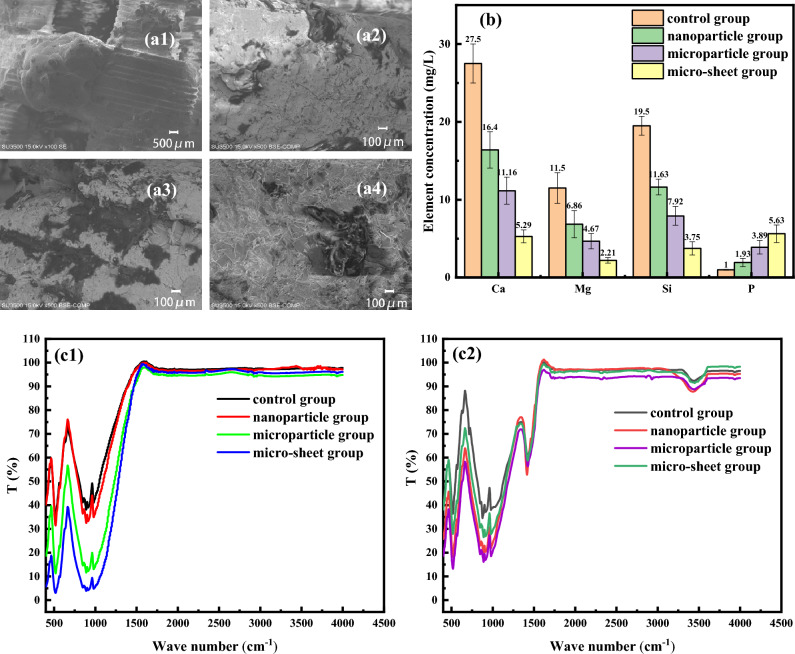
SEM micrographs for surface of the (**a1**) control group, (**a2**) nanoparticle group, (**a3**) microparticle group, (**a4**) micro-sheet group. (**b**) Concentration of Ca, Mg, Si and P element in the four groups of SBF at 2w. FTIR spectra of different micro/nano structured scaffolds before and after 7 days of degradation.

After 7 days of mineralization, the surface morphology of the four groups of scaffolds is shown in Fig. 11a1–a4. It can be observed that there is flake-like material adsorbed on the surface of the pore walls of all four groups of scaffolds. Figure 11c1,c2 shows the Fourier infrared spectra of the different micro/nano structure scaffolds before and after degradation, with the y-axis representing the transmittance of the pressed sample to infrared light at different wavelengths. The biggest difference before and after degradation is the vibration of hydroxyl ion groups in the apatite-like compound. As shown in Fig. 11c1, before degradation, only typical PO4^2−^ vibrations were detected at 501 cm^−1^, 867 cm^−1^, and 1013 cm^−1^, with no hydroxyl ion groups produced. With the presence of micro/nano structures, the transmittance of PO4^2−^ to infrared light also decreases, indicating that the amount of PO4^2−^ is increasing. After 7 days of degradation, the spectrμm shows the characteristic vibrations of hydroxyl ion groups at 1497 cm^−1^and 3570 cm^−1^, consistent with the functional groups of hydroxyapatite. As shown in Fig. 11c2. However, there was no significant difference in the transmittance of the pressed powder of the four groups of scaffolds to infrared light at these two wavelengths, indicating that the quantity of generated hydroxyl ion groups did not change much. This demonstrates that new hydroxyapatite has attached to the surface of all four groups of scaffolds, and their mineralization abilities are comparable. Therefore, the micro/nano surface structures did not alter the in vitro mineralization ability of the mesoporous bioactive glass scaffolds.

Both white hydroxyapatite and beta-tricalciμm phosphate can be degraded in vivo, producing Ca^2+^ and Mg^2+^ ions that can induce cell proliferation and accelerate bone repair by inhibiting osteoclasts from absorbing new bone tissue. Additionally, P is an essential element in the hμman body, and low levels can stimulate cell proliferation and differentiation, as well as promote bone formation. Phosphorus can form calciμm phosphate mineral in bone tissue, which is one of the main inorganic components and helps to increase bone density and strength. Furthermore, phosphorus can promote protein synthesis and cell metabolism, which helps to accelerate bone tissue repair and regeneration.

Figure 11b shows the concentration of different elements in the degradation solution of the various nano/microstructured scaffolds after 2 weeks. As shown in the figure, the concentrations of Ca, Mg, and Si decrease with the increasing thickness of the nano/microstructured scaffold. The control group blank scaffold does not contain phosphorus, while the release of P element from the nanogranular, microparticulate, and microlayered scaffolds increases with increasing structure thickness. Only the nanogranular and microparticulate scaffolds meet the requirement of P element concentration in normal hμman body fluids, which is 1.91–4.86 mg/L.

## Discussion

In this study, we put forward the concept of preparing micro/nano surfaces in porous 3D scaffolds for regeneration of osteochondral complex. Firstly, we designed the TPMS model scaffolds. Compared with other scaffolds, TPMS model scaffolds often have better mechanical properties under the same porosity. An important innovation of this research is that we combine 3D printing with hydrothermal process to prepare controllable macroporous and micro/nano surfaces in 3D porous scaffolds. In the past, traditional methods such as template method, freeze-drying and gas foaming were mainly used to prepare porous scaffolds for tissue engineering^30–33^. Compared with the traditional methods, 3D printing is a convenient and efficient method, which can effectively control the macroporous structure in the scaffold. However, the current 3D printing technology is difficult to prepare controllable micro/nano surfaces in porous scaffolds^34–37^. Due to the complex structure of 3D porous scaffolds, it is a great challenge to prepare uniform micro/nano structure surfaces on scaffolds by conventional methods. As we all know, hydrothermal method is a common and useful method to prepare micro/nano structures. In the hydrothermal process, the complex surfaces of three-dimensional porous scaffolds are in uniform contact with the reaction solution, so the surfaces of micro/nano structures are uniformly generated in the scaffolds. In addition, the micro/nano structures prepared by hydrothermal method have a high degree of size control and ordered structures^38–40^. Therefore, in this study, layered scaffolds with macroporous structure and micro/nano structure surfaces were developed by combining 3D printing with hydrothermal method.

**Figure 12:**
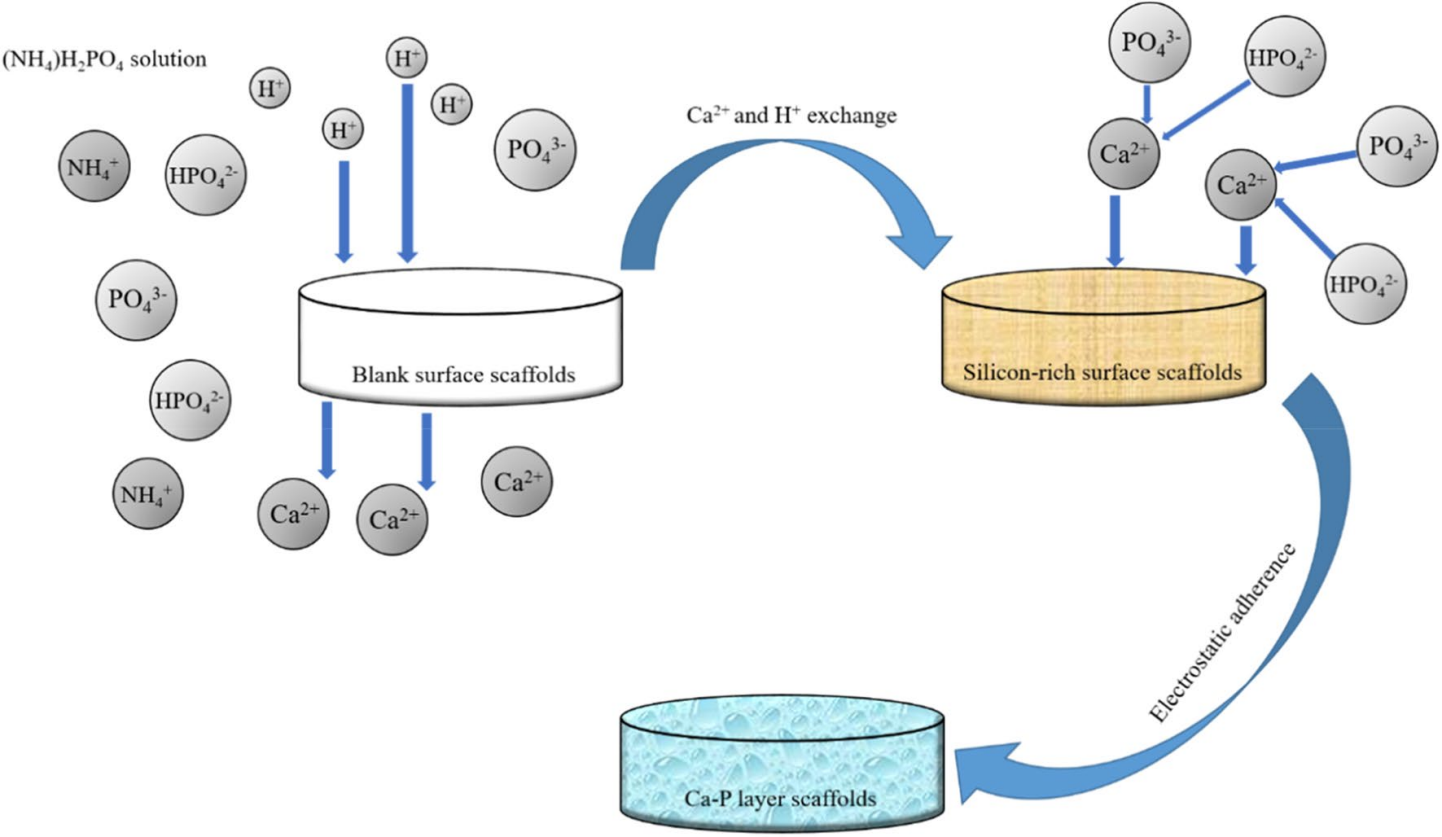
Formation process of micro/nano structure Ca-P surface.

The formation process^41^ of the nanostructure Ca-P surface can be shown in Fig. 12. Ca^2+^ on the surface of the scaffold is firstly exchanged with H^+^ in the solution, and the leaching layer rich in silanol is formed on the ceramic surface by ion exchange. In the next step, Ca^2+^ in the solution is electrostatically attracted to the newly developed negatively charged silicon-rich layer; At first, Ca^2+^ is attached to the negatively charged silicon-rich surface, and then PO_4_^3−^/HPO_4_^2−^ is adsorbed on Ca^2+^ to form a Ca-P layer. In addition, Ca-P surface morphology can be well controlled from nano-scale to micro-scale by adjusting the concentration and time of NH_4_H_2_PO_4_ solution.

In this study, the support surfaces of scaffolds grow uniformly, with micro/nano structures, with thickness of 6 μm, 24 μm and 42 μm respectively. Micro/nano surfaces have no influence on the shape of macroporous scaffolds, except that the pore diameter has decreased from 253 μm to 243 μm, 205 μm and 180 μm, and the porosity has decreased from 50.61% ± 1.23% to 49.91% ± 0.8%, 44.91% ± 0.86% and 41.95% ± 1.1%, which still meets the basic requirements of bone repair for porosity (its requirement range is between 40 and 90%) and meets the input of nutrients. The formation of Ca-P layer on the surface of scaffolds in Group nanoparticle, microparticle and micro-sheet reduced the water contact angle of scaffolds in control group from 100° to 34.1°, 30.9° and 23.8°, and the scaffolds changed from hydrophobic to hydrophilic, while the Ra also increased from 0.653 ± 0.056 to 2.5885 ± 0.1355, 3.172 ± 0.261 and 3.5305 ± 0.1375. These changes will be more conducive to cell adhesion.

Compared with control group, the compressive strength of scaffolds in nanoparticle group, microparticle group and micro-sheet group reached 59.49 ± 2.37 MPa, 64.45 ± 4.74 MPa and 86.08 ± 5.44 MPa, respectively, and the compressive strength of micro-sheet group micron-lamellae surface scaffolds is enhanced by nearly 2 times. The strains of the four groups of scaffolds when they collapse and break are 0.174 ± 0.006, 0.194 ± 0.005, 0.198 ± 0.004 and 0.199 ± 0.004 respectively. Compared with the blank surface scaffolds of control group, the strains of the other three groups of scaffolds increases slightly when they collapse, but the change among the three groups of scaffolds is not obvious, which indicates that after the blank surface scaffolds is prepared with micro/nano structures, the ductility of the scaffolds is improved to a certain extent, while the ductility of the scaffolds with the same material and similar structure is almost the same. The research shows that the in-situ grown micro/nano whiskers are pinned in the microcracks and dislocation areas, which hinders the deflection and expansion of microcracks, thus significantly enhancing the mechanical strength of porous bioceramics^42–45^.

The change of surface structure also affects the degradation of scaffolds. The quality of the scaffolds decreases with the increase of degradation time. Compared with blank surface scaffolds, the thicker the structure, the slower the degradation rate. By the 56th day, the degradation rates of the four groups of scaffolds were about 12.19% ± 0.652%, 8.82% ± 0.923%, 6.45% ± 0.537% and 2.33% ± 0.346%, respectively. According to the individual physique and the degree of osteochondral defects, it takes 6–24 months for osteochondral to be completely repaired. The degradation rates of control group, nanoparticle group and microparticle group are in line with the time range required for new bone formation rate, while the degradation rate of micro-sheet scaffolds is slow, which is not suitable for the repair of osteochondral complex. With the weight loss of the scaffold, the compressive strength of the scaffold is gradually changing. Eight weeks later, the compressive strength of the blank surface scaffold was 21.67 ± 3.33 MPa, and the compressive strength decreased by 53.53%. The compressive strength of nanoparticle, microparticle and micro-sheet scaffolds decreased to 31.46 ± 5.23 MPa, 34.15 ± 5.23 MPa and 54.77 ± 6.32 MPa, respectively. Among them, the decrease degree of nanoparticle and microparticle was 47%, and that of micro-sheet scaffolds was only 36.35%. With the increase of the thickness of the structure, the compressive strength decreases more slowly, and the gap between the compressive strength of nanoparticle, microparticle and micro-sheet scaffolds tends to decrease. In control group, the rapid degradation of scaffolds led to a sharp increase in the PH of SBF. After 8 days of degradation, the PH value reached 8.64 ± 0.06, and it was still increasing. However, the PH of scaffolds with micro/nano structures on the surfaces will also increase at the beginning of degradation, but the amplitude is obviously smaller than that of blank surface scaffolds. After 8 days, the PH values of the nanoparticle, microparticle and micro-sheet groups are 7.64 ± 0.06, 7.6 ± 0.05 and 7.44 ± 0.05 respectively, and the PH values remain in a relatively stable region in the following time, and the difference among the three groups is not big, with the highest value not exceeding 7.7. However, the PH value of the most suitable growth environment for cells in hμman body is between 7.4 and 8.2, and the alkaline environment will inhibit the growth of osteoblasts. Therefore, the scaffolds of groups nanoparticle group, microparticle group and micro-sheet group meet the PH requirements. During the in vitro experiments, no significant differences were observed in the mineralization capacity among the four scaffold groups. However, after two weeks of degradation, the concentration of P element in the micro-sheet group significantly exceeded the normal concentration in body fluids, reaching 5.63 mg/L. To sum up, The scaffolds of the nanoparticle group and microparticle group are more in line with the needs of osteochondral repair.

## Conclusion

In this study, a scaffold model with a porosity of 50% was designed to repair the defects of osteochondral complex. Combining 3D printing with hydrothermal reaction, three controllable micro/nano structured surfaces of nanoparticle, microparticle and micro-sheet were prepared in porous scaffolds. Compared with blank surface scaffolds, the surface properties, mechanical properties and degradation properties of the micro/nano structure scaffolds are improved, which provide a potential treatment method for the repair of osteochondral complex defects.

## Data Availability

All data generated or analysed during this study are included in this published article (and its supplementary information files).
